# A Joint Transcriptomic and Metabolomic Analysis Reveals the Regulation of Shading on Lignin Biosynthesis in Asparagus

**DOI:** 10.3390/ijms24021539

**Published:** 2023-01-12

**Authors:** Junying Ma, Xiaoyan Li, Maolin He, Yanwen Li, Wei Lu, Mengyao Li, Bo Sun, Yangxia Zheng

**Affiliations:** College of Horticulture, Sichuan Agricultural University, Chengdu 611130, China

**Keywords:** asparagus, shading treatment, lignin biosynthesis, transcriptomic, metabolomic, combined analysis

## Abstract

Asparagus belongs to the Liliaceae family and has important economic and pharmacological value. Lignin plays a crucial role in cell wall structural integrity, stem strength, water transport, mechanical support and plant resistance to pathogens. In this study, various biological methods were used to study the mechanism of shading on the asparagus lignin accumulation pathway. The physiological results showed that shading significantly reduced stem diameter and cell wall lignin content. Microstructure observation showed that shading reduced the number of vascular bundles and xylem area, resulting in decreased lignin content, and thus reducing the lignification of asparagus. Cinnamic acid, caffeic acid, ferulic acid and sinapyl alcohol are crucial intermediate metabolites in the process of lignin synthesis. Metabolomic profiling showed that shading significantly reduced the contents of cinnamic acid, caffeic acid, ferulic acid and sinapyl alcohol. Transcriptome profiling identified 37 differentially expressed genes related to lignin, including *PAL*, *C4H*, *4CL*, *CAD*, *CCR*, *POD*, *CCoAOMT*, and *F5H* related enzyme activity regulation genes. The expression levels of *POD*, *CCoAOMT*, and *CCR* genes were significantly decreased under shading treatment, while the expression levels of *CAD* and *F5H* genes exhibited no significant difference with increased shading. The downregulation of *POD*, *CCoAOMT* genes and the decrease in *CCR* gene expression levels inhibited the activities of the corresponding enzymes under shading treatment, resulting in decreased downstream content of caffeic acid, ferulic acid, sinaperol, chlorogenic acid and coniferin. A significant decrease in upstream cinnamic acid content was observed with shading, which also led to decreased downstream metabolites and reduced asparagus lignin content. In this study, transcriptomic and metabolomic analysis revealed the key regulatory genes and metabolites of asparagus lignin under shading treatment. This study provides a reference for further understanding the mechanism of lignin biosynthesis and the interaction of related genes.

## 1. Introduction

Light is the energy source for plant growth and development. The photosynthetic intensity, photosynthetic area and photosynthetic time determine the amount of photosynthate accumulation. In contrast, shading greatly reduces the light intensity, thus decreasing material accumulation, reducing stalk fullness, and affecting the mechanical strength of the plant stem. Lignin is deposited in the secondary cell wall along with cellulose and hemicellulose [1], and cross-linking with cellulose and hemicellulose contributes to stem stiffness and mechanical strength [2]. The mechanical strength of the stem is closely related to the lignin content in the stem cell wall [3]. At the same time, lignin is a photosensitive phenolic compound, and the change in light environment directly affects lignin synthesis. In some previous research, 60% shading reduced lignin deposition in rice stem sclerenchyma and vascular bundle (VB) cells [4]. A gradual decrease in lignin content in soybean stems was also observed with the increase in shading intensity [5]. Shading significantly impacts lignin biosynthesis by affecting its metabolic activity [6]. In conclusion, the regulation of light intensity can significantly affect lignin accumulation in plants.

Lignin is an essential component of the plant cell wall. Its synthesis is divided into three steps. Firstly, cinnamic acid is generated from phenylalanine deamination, and then the lignin monomer is synthesized through a series of hydroxylation reactions (*C4H*, *C3H*, *F5H*, *4CL* and *HCL*), 0-methylation reactions (*COMT* and *CCoAOMT*), and reduction reactions (*CCR* and *CAD*). Finally, the lignin monomers associated with three major types of monophenols are polymerized to lignin deposited in the secondary cell wall [7,8,9,10]. Numerous studies have investigated the effect of single or multiple lignin biosynthesis genes on lignin content. For example, the lignin content of *PAL* gene mutants showed a 20–25% decrease compared with the wild type [11]. The functions of *CAD* and *CCR* were disrupted in Arabidopsis mutants, and a 50% reduction in lignin content was observed compared with the wild type, accompanied by the male sterile phenotype [12]. Furthermore, inhibiting the expression of the *CCR* and *COMT* genes significantly changed the lignin content and composition of ryegrass and improved its digestibility [13]. Transgenic alfalfa expressing the antisense structure of *HCT* had significantly lower lignin content, and significant changes in lignin composition were found, manifesting as obvious growth retardation, biomass decline and flowering delay [14]. The expressions of the *CAD* and *COMT* genes related to lignin biosynthesis and lignin content in leaves of inbred maize lines were significantly positively correlated with drought tolerance [15]. Expression of the *CCR* gene in maize [16] root elongation zone and *CCoAOMT* gene in the soybean [17] root elongation zone were significantly enhanced under drought stress. Increased lignin content in this region inhibits root cell wall elongation, limiting root water loss and promoting water transport to the elongation zone. Therefore, lignin plays a crucial role in plant growth, development and environmental adaptability. 

Asparagus (*Asparagus officinalis* L.) belongs to the Liliaceae family. It is used for culinary and medicinal purposes due to its high nutritional and medicinal value, containing carbohydrates, protein, free amino acids, vitamins, minerals and dietary fiber, etc. [18]. Asparagus is rich in flavonoids [18], phenols, saponin compounds [19,20] and other bioactive substances (such as phenolic acids, protopurines, carotenoids and oligosaccharides, etc.) [21]. Therefore, asparagus has antioxidant, antibacterial, antitumor and anticancer properties [22,23]. The temperature rises above 30 °C in summer, and the base and outer bark of the tender stem are prone to fibrosis, while the scale of the stem end is easy to spread, aggravating the lignification degree and reducing the quality. This greatly reduces the edible and commodity value of asparagus. ‘Fengdao No. 2′ was used as plant material, and the distribution and content of asparagus lignin were evaluated by physiological methods and histochemical staining. In addition, transcriptomic and metabolomic analysis were conducted to elucidate the regulation mechanism of shading effect on lignin biosynthesis of asparagus stems, which provided certain theoretical bases for cultivation methods to improve the growth and quality of asparagus in the summer’s high-temperature season. Numerous studies have shown that shading reduces plant lignin. Nevertheless, the influence of shading on asparagus lignin has not been reported.

## 2. Results

### 2.1. Influence of Shading on the Morphological Characteristics of Asparagus Tender Stem

**Figure 1:**
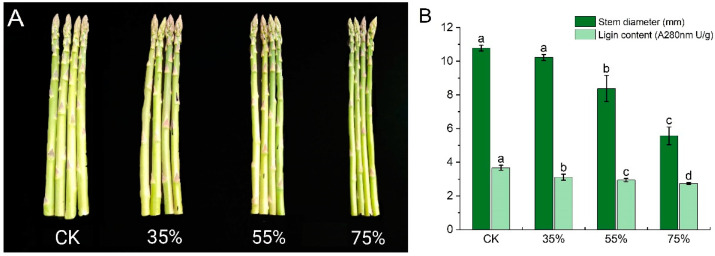
Effect of shading on asparagus tender stem. (**A**) Phenotypic characteristics of asparagus tender stem under different shading treatments. (**B**) Changes of lignin content in asparagus tender stem under different shading treatments. Each bar represents the mean ± SD. *T* test at a significance level of 0.05 (*p* < 0.05). Different letters (a, b, c, d) indicate significant differences, and the same letter indicates no significant differences.

The asparagus was harvested when it reached the commercial standard length of about 35 cm, and its diameter was measured (Figure 1A). Compared with CK, the stem diameter of the asparagus’ tender stem showed a downward trend with the increase in shading intensity (Figure 1B), with a significant difference between treatments. Compared to the CK group, the 35% shading, 55% shading and 75% shading groups resulted in a 4.08%, 22.26%, and 48.33% decrease in tender stem diameter, respectively. Therefore, the asparagus tender stem diameter was significantly affected by shading. Moreover, compared with the CK group, the lignin content of the asparagus tender stem decreased with increasing shading intensity, showing significant differences between treatment groups (Figure 1B).

### 2.2. Histochemical Staining Analysis of Lignin Morphological Distribution in Asparagus under Shading

**Figure 2:**
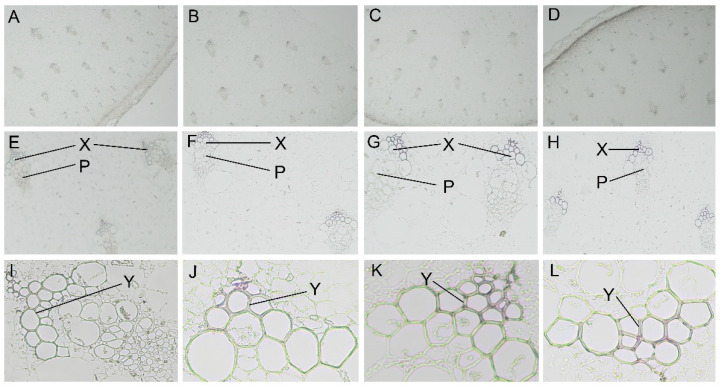
Lignin staining changes of asparagus under different shading treatments. (**A**) Full light ×40; (**B**) 35% shading ×40; (**C**) 55% shading ×40; (**D**) 75% shading ×40 (**E**) no shading ×100; (**F**) 35% shading ×100; (**G**) 55% shading ×100; (**H**) 75% shading ×100 (**I**) no shading ×400; (**J**) 35% shading ×400; (**K**) 55% shading ×400; (**L**) 75% shading ×400; (P) phloem; (X) xylem; (Y) cell walls (×40, ×100, and ×400 represent 40×, 100×, and 400× microscope magnification, respectively).

The morphological effect of shading on lignin accumulation in asparagus was investigated. A quarter of the cross-sections of asparagus shoots was stained with phloroglucinol to detect the distribution of lignin. The lignin composition was stained purplish red, while the background was colorless or the color of the tissue itself. As shown in Figure 2, lignin distribution was obvious under phloroglucinol staining, and lignin was mainly deposited in the xylem region. In the absence of shading, most cells were lignified and closely arranged, with a large proportion of red-stained areas. However, with the increasing shading, the number of lignified cells gradually decreased, and the cell arrangement also gradually loosened. The red staining area gradually became smaller and lighter, reaching the minimum and lightest at 75% shading, where the cell arrangement was the worst. The lignin content under different treatments was consistent with Figure 1B.

### 2.3. Transcriptome Sequencing and Whole Gene Expression Analysis

**Figure 3:**
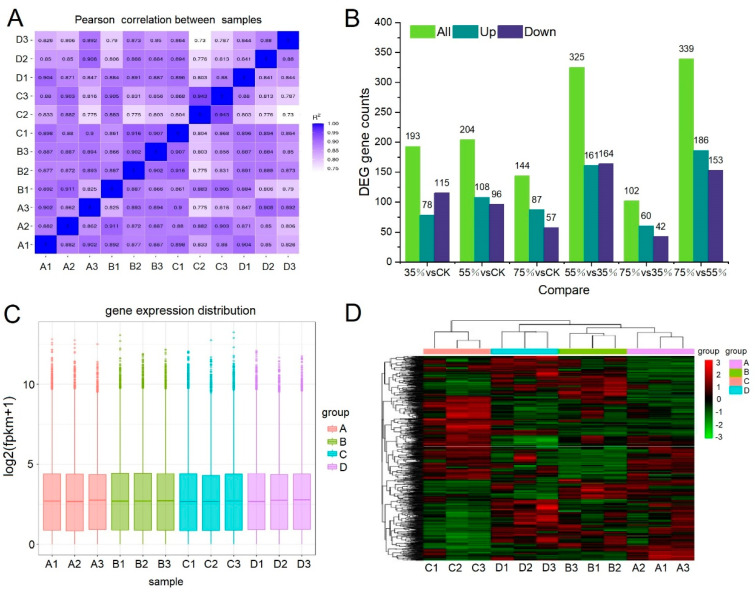
Gene expression analysis of asparagus after shading treatment. (**A**) Pearson correlation coefficient under different shading treatments, where A: full light. (**B**) Number of DEGs under any two different treatments. The number of upregulated and downregulated genes is shown in grey and green bars, respectively. (**C**) Box plots of gene expression levels in different samples. (**D**) Clus-tering heat map between groups.

The gene changes of asparagus in response to shading were studied by RNA-seq. A total of 522,244,482 original reads were generated from all samples, and 489,020,094 high-quality clean reads were screened, detecting a total of 35,351 transcripts. After calculating the expression value (FPKM) of all genes in each sample, the distribution of gene expression levels in different samples was shown by a box graph (Figure 3B). The box graph of each region also presents five statistics (top to bottom are maximum, upper quartile, median, lower quartile and minimum). The correlation of gene expression levels between samples can be used to measure the reliability of experiments and the rationality of sample selection. The closer the correlation coefficient is to 1, the higher the similarity of expression patterns between samples. The intra-group and inter-group correlation coefficients of samples were calculated according to the FPKM values of all genes in each sample, and a heat map was generated. The latter visually displays the sample differences between groups and sample duplication within groups. A higher correlation coefficient between samples indicated a closer expression pattern. The sample correlation heat map is shown below (Figure 3A). In any given shading treatment, the results showed that plants treated with A showed a higher gene expression level correlated with plants treated with B and D, indicating that the gene expression level was similar to that of plants treated with B and D without the influence of shade intensity. The lower the correlation coefficient between the plants treated with C and those treated with A, B and D, the higher the difference of their expression pattern under C treatment. Figure 3C displays the differentially expressed genes of each comparison combination. Compared with CK, 193, 204 and 144 differentially expressed genes (DEGs) were found in the 35% shading, 55% shading and 75% shading groups. Among the six compared pairs, the number of differentially expressed genes was the least in 75% vs. 35%, indicating that the gene expression pattern of asparagus was similar at 35% shading and 75% shading. In addition, the number of differentially expressed genes was the highest in 55% vs. 35% and 75% vs. 55%, indicating that 55% shading triggered the expression of specific genes. A hierarchical clustering heatmap was generated to view the expression patterns of the four shade treatments (Figure 3D). The results indicated good repeatability within the three replicates of each group, with a significant difference between groups.

### 2.4. DEGs Analysis

**Figure 4:**
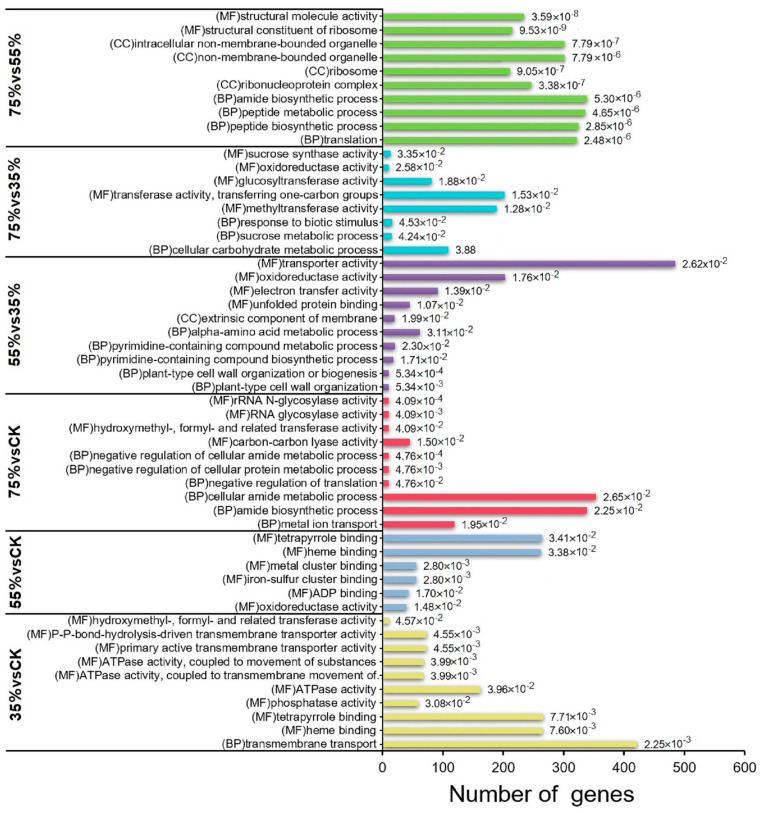
Top 10 GO enrichment terms with highly significant *p* values (≤0.05) in each pair of comparisons.

Gene ontology (GO) annotation was conducted on all comparison pairs to further analyze the functions of DEGs. Figure 4 shows the top 10 enriched terms belonging to the biological process (BP), cellular component (CC) and molecular function (MF). Most DEGs were enriched in biological processes (BP) and molecular functions (MF). In the BP, the inter-group comparison of 75% vs. CK, 55% vs. 35%, 75% vs. 35%, and 75% vs. 55% were significantly enriched in translation, plant-type cell wall organization, cell carbohydrate metabolism, sucrose metabolism, amino acid metabolism, peptide biosynthesis, amide biosynthesis, and peptide and thin cytoamide metabolism. Among them, plant cell wall organization, sucrose metabolism and amino acid metabolism were closely related to the accumulation of glucose and lignin. In the MF, the comparison of 35% vs. CK and 55% vs. CK showed that phosphatase, ADP and ATPase activities were significantly enriched terms. In contrast, 75% vs. CK, 55% vs. 35%, 75% vs. 35%, and 75% vs. 55% showed significant enrichment in transferase activities such as methyl, glucosaccharide, hydroxymethyl and formyl, oxidoreductase activities and sucrose synthase activities. For the CC category, the GO terms significantly enriched in the 75% vs. 55% and 55% vs. 35% comparison groups were external components of the membrane, ribonucleoprotein complex and organelles. Through the MF and CC analyses, no obvious changes were found at 35% shading treatment, while significant changes were observed at 55% and 75% shading treatment. These findings indicated that shading exerted a certain effect on asparagus.

### 2.5. Metabolome Analysis

**Figure 5:**
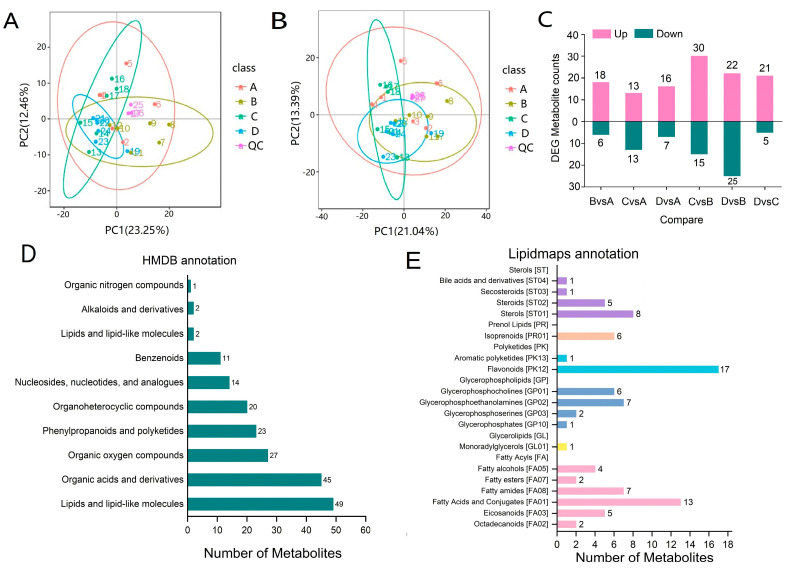
Identification of metabolites of asparagus under shading treatment. (**A**) PCA in negative ion modes. (**B**) PCA in positive ion modes. (**C**) Metabolites change under different shading treatments. (**D**) Metabolites classified by HMDB database. (**E**) Metabolites classified by lipid profile database.

The metabolites of asparagus during shading were assessed by UHPLC-MS/MS. In total, 321 metabolites were detected in the positive ion mode, and 500 metabolites were detected in the negative ion mode. The largest difference was observed between D and B, which produced the largest number of differential metabolites (*n* = 47), followed by C vs. B (*n* = 45). However, the comparison group with the least difference was D vs. A, with the least amount of differential metabolites (*n* = 23). In total, 27 samples were plotted in the PCA chart using different colors to represent the shading treatment. For the negative ion samples, the PCA multispace for axes 1 and 2 explained about 35.71% of the total variation (Figure 5A), and for the positive ion samples, the PCA multispace for axes 1 and 2 explained about 34.43% of the total variation (Figure 5B). However, the samples of C and D are almost completely overlapped, showing high similarity. Samples B, A and C and D were almost completely separated. In addition, the quality control (QC) samples on the PCA analysis chart were clustered into five groups, and the separation was obvious. HMDB and Lipidmaps databases were used to label the identified metabolites. In the HMDB database, a total of 194 metabolites were classified into 10 categories (Figure 5C,D), of which the most enriched types were lipids and lipid molecules (49 metabolites), followed by organic acids and derivatives (45 metabolites), phenylpropanoids and polyketides (23 metabolites), and organooxygen compounds (27 metabolites). In the Lipidmaps database (Figure 5E), the most diverse type was lipoacyl (6 classes, 33 metabolites), followed by glyceropholipids (4 classes, 16 metabolites), and polyketides (2 classes, 18 metabolites).

### 2.6. Transcriptome and Metabolome Pathway Enrichment Analysis

**Figure 6:**
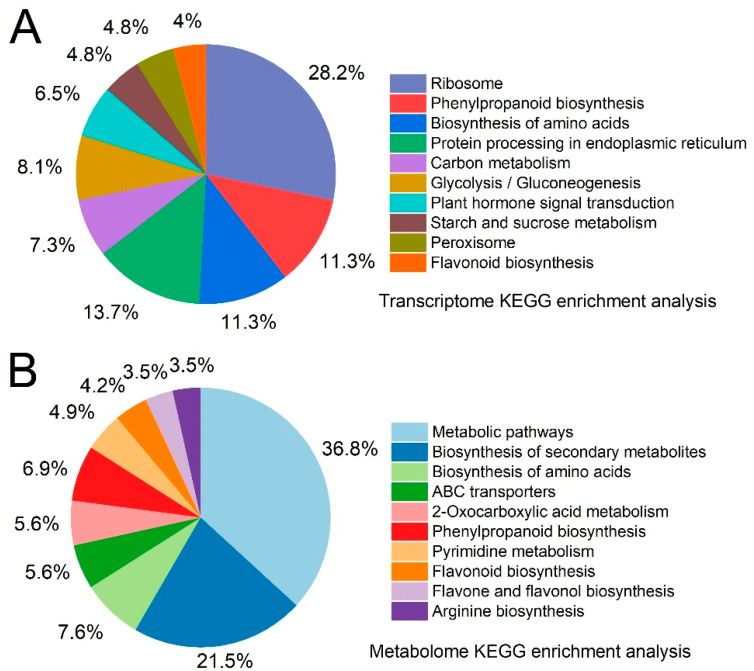
Enrichment analysis of the top 10 KEGG pathways associated with transcriptome and metabolome. (**A**) Transcriptome. (**B**) Metabolome. The percentage indicates the number of differential metabolites or differential genes/metabolites or genes annotated by the pathway enriched by the pathway.

KEGG enrichment analysis was performed to identify the significantly enriched metabolic pathways associated with DEGs and differential metabolites. Figure 6 shows the top 10 metabolic pathways with a differentially enriched transcriptome and metabolome in each comparison. The highly expressed pathways in the transcriptome and metabolome include amino acid metabolism, flavonoid synthesis, phenylpropanoid biosynthesis and other pathways. The phenylpropanoid biosynthesis pathway is particularly relevant to the study of lignin synthesis.

### 2.7. Transcriptome and Metabolome Reveal Shading Analysis of Lignin Accumulation in Asparagus

**Figure 7:**
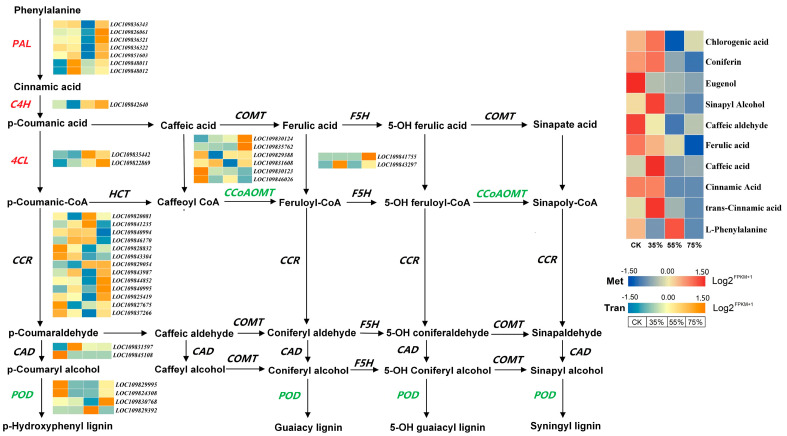
Heat map of lignin metabolites and gene expression of asparagus under shading treatment. Red indicates upregulation, and blue indicates downregulation. The expression decreased gradu-ually from red to blue, and the data were calculated using FPKM.

The synthesis and decomposition of lignin in plants are usually related to the activities of enzymes involved in lignin synthesis. In order to determine the molecular mechanism of lignin accumulation in asparagus, the genes involved in lignin biosynthesis were screened from the asparagus transcriptome, revealing a total of 37 lignin-related DEGs (Figure 7). The enzymes encoded by these genes include PAL (phenylalanine lyase), C4H (cinnamate-4-hydroxylase), 4CL (4-coumarate-CoA ligase), CAD (cinnamyl alcohol dehydrogenase), CCR (cinnamyl CoA reductase), POD (peroxidase), CCoAOMT (caffeyl-Coa-oxy-methyltransferase), and F5H (ferulic acid 5-hydroxylase). Compared with normal light, shading treatment exerted significant effects on key enzyme genes in the lignin synthesis pathway of asparagus, upregulating the *PAL*, *C4H* and *4CL* genes. In contrast, the expressions of *CCoAOMT* and *POD* genes were downregulated, and shading inhibited the expression of *POD* and *CCoAOMT* genes. Some homologous genes showed different expression patterns under shading conditions. For example, genes encoding CCR enzymes, *LOC 109829054*, *LOC109827675* and *LOC109820081* genes, had the lowest expression levels in 35% shading treatment. *LOC109841235*, *LOC109840994* and *LOC109846170* genes had the lowest expression in 75% shading treatment, while *LOC109843987* and *LOC109844852* genes had the lowest expression in 55% shading treatment. These results suggest that *CCR* is involved in the regulation of lignin biosynthesis in asparagus. However, *CAD* and *F5H* showed no significant change. The heat map also illustrates that the expression levels of most genes reached the lowest at 55% shading treatment. The metabolome detected 10 metabolites related to lignin (Figure 7), namely phenylalanine, trans-cinnamic acid, cinnamic acid, caffeic acid, dihydroxycinnamic acid, ferulic acid, dihydroxycinnamic aldehyde, sinapinol, eugenol, coniferin, and chlorogenic acid. Cinnamic acid and caffeic acid were significantly upregulated in 35% shading treatment and significantly downregulated with the increase in shading degree. The metabolites of dihydroxycinnamic acid, ferulic acid, dihydroxycinnamic aldehyde and sinapirol were significantly downregulated under shading treatment, except for phenylalanine, which showed significantly higher levels in 55% shading treatment compared to other treatments. The expression level of other metabolites were significantly decreased in the 55% and 75% shading groups.

**Figure 8:**
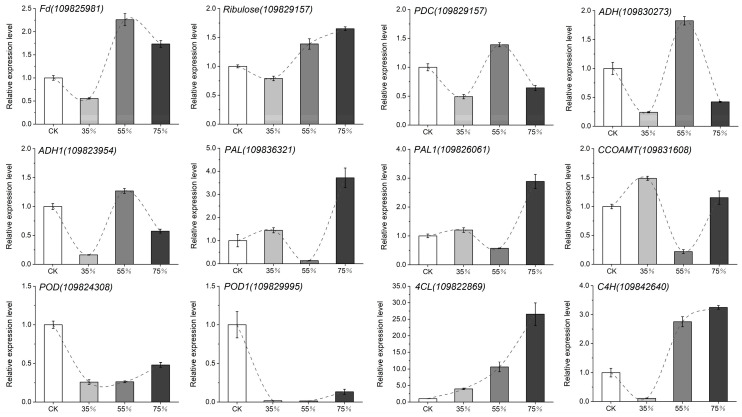
qRT-PCR analysis of asparagus under shading treatment. Relative expression levels of, *Fd*, *Ribulose*, *PDC*, *ADH*, *ADH1*, *PAL*, *PAL1*, *CCOAMT*, *POD*, *POD1*, *4CL* and *C4H*. Each column represents the mean ± SD. *T* test at the significance level of 0.05 (*p* < 0.05).

Twelve genes related to photosynthesis (*Ribulose, Fd*), glucose metabolism (*FBA*, *PDC*, *ADH*), phenylalanine, tyrosine (*DAHP*) and lignin synthesis (*PAL*, *C4H*, *4CL*, *POD*, *CCOAMT*) pathways were selected for verification (Figure 8). After shading treatment, a significant downregulation of about 1 time and 0.25 times was observed in the photosynthetic genes *Fd* and *Ribulose*. The expression levels of glucose metabolism pathway genes *PDC*, *ADH* and *ADH1* reached the lowest and were significantly downregulated by more than two-, four- and six-fold in the 35% shading group, respectively. The expression level reached the maximum in the 55% shading group. The expression levels of lignin pathway genes *PAL1*, *PAL* and *CCOAMT* reached the lowest and were significantly downregulated by more than 1.5, 8 and 5 times under 55% shading treatment, respectively. However, the expression of *POD* and *POD1* genes was significantly downregulated by 4-fold and 10-fold after 35% shading and 55% shading. No significant difference was observed between these two treatments. These results are consistent with the transcriptome studies.

## 3. Discussion

Some studies found that the plant stem diameter was reduced by shading treatment [24,25]. This study also found significantly reduced stem diameter and lignin content of asparagus under shading conditions compared with normal light. Moreover, many studies have shown that the *PAL*, *C4H*, *4CL*, *CAD*, *CCR*, *POD*, *CCoAOMT* and *F5H* genes play essential roles in lignin biosynthesis pathways [26,27,28]. Inhibition of *CCoAOMT* enzyme activity resulted in significantly reduced lignin content in transgenic tobacco [29,30]. Reduced lignin synthesis was observed after the *CCR* gene was knocked out in Arabidopsis [31]. In transgenic poplars, the decrease in lignin content was closely related to the decrease in POD enzyme activity in leaves [32].

Lignin is sensitive to changes in light intensity [33]. High light intensity led to increased lignin content in the stem of soybean [34], while low light intensity resulted in decreased lignin content in the stem of rice [4], maize [35], soybean [33,36,37] and tobacco [38]. In this study, the *PAL*, *C4H*, *4CL*, *CAD*, *CCR*, *POD*, *CCoAOMT* and *F5H* genes related to lignin synthesis in asparagus were all affected by the shading conditions. Especially, *POD* and *CCoAOMT* genes were significantly downregulated, suggesting that these two genes played key roles in lignin synthesis in asparagus under shading, resulting in reduced lignin content. A large number of studies have shown that the expression of *PAL*, *CCoAOMT*, *CCR*, *COMT* and *CAD* genes is significantly inhibited in rice stems under 60% shading, also resulting in lignin reduction [4]. Compared with the control group (without shading), shading downregulated the expression of lignin biosynthesis genes such as *PAL*, *C4H*, *4CL*, *CCOAOMT*, *COMT*, *CCR*, *CAD* and *LAC* in soybean stems [37]. A similar study was performed on the stem of herbaceous peony, revealing that shading caused a significant inhibition of the *CCoAOMT* gene, followed by *POD* and *CCR* genes, which significantly reduced the stem lignin content [24]. Therefore, the reduction of lignin content in asparagus under shading treatment was speculated to be closely related to the decrease in *CCR*, *POD* and *CCoAOMT* gene expression. In this study, the *PAL*, *C4H* and *4CL* genes were significantly upregulated under shading treatment. Single or independent genes may respond to different abiotic stresses, and their expression is controlled by time and space [39,40]. In addition, *PAL* enzyme activity was reduced by 30% with antisense RNA technology, but the lignin content showed no significant change [41]. This indicates that the individual *PAL*, *C4H* and *4CL* genes exert uncertain effects on lignin content. Therefore, we concluded that the *PAL*, *C4H* and *4CL* genes in this study had little effect on lignin content in asparagus under shading conditions.

Carbon flow from the shikimic acid pathway directly reaches various branches of phenylpropane metabolism, including the lignin synthesis pathway [42]. In this process, cinnamic acid is hydroxylated to produce p-coumaric acid, which is then converted into caffeic acid, ferulic acid, and sinapic acid through a series of reactions. These five acids are important intermediate metabolites in lignin synthesis [2]. This study demonstrated a significant upregulation in the expressions of cinnamic acid and caffeic acid in asparagus under 35% shading treatment, which was then significantly downregulated as shading increased. These findings indicated that the metabolic pathway of phenylpropane was strengthened under shading treatment. Lignin monomer synthesis consists of two pathways, one is from phenylpropane to cinnamic acid, p-coumaric acid, coumaryl CoA, coumaraldehyde, followed by the formation of coniferol and sinrosinol, and the other is from coumaric acid to coumaric CoA, caffeyl CoA, or caffeic acid, ferulic acid, sinapic acid, coniferol, sinapitol, and finally polymerized into lignin monomers [43]. In this study, the contents of cinnamic acid, caffeic acid, ferulic acid and sinapirol decreased with the increase in shading, indicating that the second metabolic pathway plays a major role in asparagus. These five metabolites are closely related to the H lignin monomer, G lignin monomer and S lignin monomer, and thus, they eventually lead to the reduction of lignin content. When plant photosynthesis is weak or inhibited, low concentrations of intermediate metabolites in the shikimic acid pathway reset the entire phenylpropanoid metabolic pathway to generate more flavonoids, anthocyanins, some volatile substances, or to synthesize some proteins to resist abiotic stress [44,45]. In this study, the photosynthesis of asparagus was inhibited by shading, resetting the metabolic pathway of phenylpropane. Furthermore, cinnamic acid may generate many other metabolites, such as ubiquitin and other terpenoid quinones.

## 4. Conclusions

In this study, various biological methods were used to investigate the mechanism of shading affecting the lignin accumulation pathway in asparagus. The downregulation of the *POD* and *CCoAOMT* genes and the decrease in *CCR* gene expression resulted in decreased activities of the corresponding enzymes. Therefore, reduced contents of caffeic acid, ferulic acid, sinapirol, chlorogenic acid and coniferin were observed downstream. Moreover, the upstream content of cinnamic acid significantly decreased with increased shading, also resulting in the decrease in the content of downstream metabolites. Finally, the lignin content of asparagus was reduced. The synthesis and accumulation of lignin are jointly determined by genes in various metabolic pathways. The *CCR*, *POD* and *CCoAOMT* genes are the key genes for lignin synthesis in asparagus. Cinnamic acid, caffeic acid, ferulic acid and sinapirol are also the key intermediate metabolites affecting lignin content. This study elucidates the mechanism of lignin formation in asparagus under shading treatment, identifying the genes and metabolites related to lignin biosynthesis. These results indicate that lignin biosynthesis in asparagus is regulated by key enzyme genes and metabolites and provide a reference for further understanding the mechanism of lignin biosynthesis in plants and the interaction of related genes.

## 5. Materials and Methods

### 5.1. Asparagus Material

The asparagus variety ‘Fengdao No. 2′ was used in this study. The asparagus base was located in Enyang District, Bazhong City, Sichuan Province, China (31°86′ N, 106°74′ E) and was shaded on July 2021, including four different shading treatments (full light, 35% shading, 55% shading, 75% shading). After 21 days, 50 healthy asparagus plants with the same growth status and without pests and diseases were selected for morphological characterization, physiological, transcriptome and metabolome sampling from each treatment group. Three biological replicates were set for physiological and transcriptome data, and six biological replicates were set for metabolomic data. After sampling, the samples were rapidly frozen in liquid nitrogen and stored at −80 °C.

### 5.2. Determination of Stem Diameter and Lignin Content

The stem diameter was measured with vernier calipers. Lignin content was determined according to the method described by Morris [46]. The absorbance value was measured at 280 nm, and lignin content was expressed as A280 nm U/g.

### 5.3. Histochemical Staining of Lignin Morphology Distribution

Five healthy asparagus plants with the same growth condition and no pests and diseases were collected from each treatment group. The middle part of the stem was cut into 2 cm thick pieces and placed into 50 mL 50% FAA fixing solution. The samples were fixed at 4 °C for 24 h and stained with phloroglucinol, giving a red color to the lignin composition while the background retained its natural color. Photographs were taken within 3 min, and the results were saved.

### 5.4. Transcriptome Sequencing and Annotation

Total RNA was extracted using the Trizol method according to the manufacturer’s instructions. The Agilent 2100 bioanalyzer was used to accurately detect the integrity and total amount of RNA. The cDNA libraries were sequenced on the Illumina HiSeq 4000 sequencing platform (Biomarker Technologies Co., Ltd., Beijing, China). FPKM (fragments per kilobase of transcript per million fragments mapped) was used as an index to measure the level of transcripts or gene expression. DESeq2 (version 1.20.0) was used to analyze the differential expression between sample groups. Transcriptome sequencing was completed by Nuohe Gene Technology Co., Ltd., Beijing, China.

### 5.5. Extraction and Detection of Metabolites

After transferring 100 mg asparagus sample to a 1.5 mL centrifuge tube, the supernatant was collected and analyzed by UHPLC-MS/MS [47]. The off-machine data (RAW) files were imported into CD 3.1 search software for processing, and the identification and relative quantitative results of metabolites were obtained. The default criteria for differential metabolite screening were VIP > 1, *p* value < 0.05 and FC ≥ 2 or FC ≤ 0.5.

### 5.6. RT-qPCR Validation

The total RNA of the asparagus samples was extracted with an RNA extraction kit (Ai Weidi Biotechnology Co., Ltd., Shenzhen, China). CK, 35% shading, 55% shading and 75% shading cDNA were used as templates, and specific primers were designed using primer Premer6 (Supplementary Materials Table S1). Relative gene expression was normalized with the reference gene (*X66875.1*) and calculated with the 2^-ΔΔCt^ method [48].

## Data Availability

Transcriptional and metabolic data were generated by Nuohe Gene Technology Co., Ltd., and physiological and anatomic metabolic data were measured by the authors themselves.

## Supplementary Materials

The following supporting information can be downloaded at: https://www.mdpi.com/article/10.3390/ijms24021539/s1.
